# What Will Happen to This Dog? A Qualitative Analysis of Rehoming Organisations' Pre-adoption Dog Behaviour Screening Policies and Procedures

**DOI:** 10.3389/fvets.2021.796596

**Published:** 2022-01-14

**Authors:** Karen E. Griffin, Elizabeth John, Tom Pike, Daniel S. Mills

**Affiliations:** ^1^School of Life Sciences, College of Science, University of Lincoln, Lincoln, United Kingdom; ^2^Department Population Health Sciences, Unit Animals in Science and Society, Animal Behaviour, Faculty of Veterinary Medicine, Utrecht University, Utrecht, Netherlands; ^3^The Dog Rehoming Project, Irvine, CA, United States; ^4^College of Science, University of Lincoln, Lincoln, United Kingdom

**Keywords:** behaviour evaluation, dog assessment, rehoming, rehoming organisation, shelter

## Abstract

Rehoming organisations often undertake some type of behaviour evaluation to determine dogs' suitability for rehoming and/or the type of suitable home. Assessments can carry considerable weight in determining dogs' fates. Although evaluation of the validity and reliability of any test is important, a more fundamental consideration is if the nature of the information sought and the weight given to this in organisations' decision making is of more than anecdotal value. Therefore, this study's aim was to conduct a qualitative analysis of organisations' pre-adoption dog behaviour screenings and potential justifications, comparing this with the available scientific evidence. A written enquiry was sent electronically to rehoming organisations in the UK and US from February 2016-July 2017. Of 73 respondents, the majority conducted assessments for all dogs. Using a thematic analysis, nine themes and 71 sub-themes emerged concerning the types of information respondents aim to gather from assessments. The majority of respondents used, at least partially, *pass/fail* scoring, i.e., certain outcomes would lead to dogs being deemed unadoptable. Forty-one sub-themes and one theme were identified as potentially leading to a dog being deemed unadoptable. The evidence base for these factors was identified from the scientific literature relating to: increased risk for relinquishment, impact on a dog's quality of life, and human safety risk. Evidence supported 10 factors: “aggression towards people”, “aggression towards cats or other animals”, “aggression towards dogs”, “biting or snapping”, “resource guarding”, “activity level or exercise needs”, “destructiveness”, “housetrained”, “fearfulness”, and “knowledge of basic commands and/or general training”. Of those, seven were associated only with relinquishment risk, two (“resource guarding”, “knowledge of basic commands”) with human safety risk, and one (“fearfulness”) with both. Thus, for >85% of characteristics organisations deemed important for dogs' adoptability, scientific evidence to support this is lacking. More research is needed to investigate the value of behaviour assessments, especially concerning the assessment of factors that could pose a public safety risk. However, given the current lack of scientific support for many decisions regarding dogs' rehoming suitability and recognised pressure on resources, it is suggested that organisations should focus on pre-adoption adopter education and post-adoption support.

## Introduction

Animal shelters and rehoming organisations commonly use temperament, behaviour, or personality tests to screen dogs pre-adoption. Mornement et al. (1) reported that organisations undertake such assessments for two main purposes: to reduce liability and to improve prospects of a successful rehoming. A third reason why some organisations conduct assessments, which is being increasingly recognised, is to identify which dogs require additional training or rehabilitation prior to rehoming. There is potentially an additional key reason for conducting such assessments: an attempt to ensure good welfare and quality of life for the dog in their new home. In the second case, to improve prospects of a successful rehoming, organisations tend to focus on trying to get some form of match between adopter and dog with the hope that it will lead to a successful placement. For the dog this is often done by gathering as much information as possible during a behaviour assessment (e.g., behaviour around small children, behaviour around other dogs, and behaviour around food) (1–3).

The formality and standardisation by which organisations behaviourally assess dogs can vary widely (1), but regardless of the formality of the assessment method used, they often focus on predicting behaviour in situations or settings that a dog is likely to encounter in a home environment (1, 4, 5). This is largely accomplished by recording the responses of dogs to a variety of stimuli, and proxies may be used for this purpose (e.g., rubber hand, child-like doll) (3, 6). Additionally, while some organisations may employ widely used and well-known assessments [e.g., Match Up Behaviour Evaluation (6)], others may use an assessment protocol they have developed in-house, while others may use a totally unstructured method of assessment. One of the more structured dog-adopter matching processes is the ASPCA's Meet Your Match™ Canine-ality™ adoption program, which aims to successfully match dogs with potential adopters by evaluating five aspects of a dog's behaviour (friendliness and sociability, playfulness, energy level and ability to focus, motivation, and “people manners”) (4, 7). Potential adopters also complete a survey corresponding to what is evaluated in the dog assessment; they are then classified into one of three groups and assigned a colour. The colour categories to which dogs are assigned are purportedly based on their assessment score and on the evaluator's determination of a dog's source of motivation (internal, external, or social) during the assessment (4). Within each colour category they are divided a further three times so that each one has a Canine-ality™ name and description. When a potential adopter is seeking a dog, they find one that has a matching colour to their own, the assumption being that the dogs coded with the same colour will be a good fit based on what their lifestyle is and what characteristics they would prefer in a dog (4).

A considerable body of research in several countries has focused on examining these tests, often in terms of their reliability and validity, [e.g., (3, 6, 8, 9)], although recent reviews have heavily criticised the predictive value of any such test (10, 11). However, these are not the only evaluations undertaken and there is a lack of information on what information organisations actually aim to gather from pre-adoption dog assessments, regardless of the screening tool used. Gaining insight into this aspect of dog assessments is an initial step towards understanding what information about a dog is most useful in both rehoming a dog and increasing the likelihood that it results in a successful placement. By having an understanding of the types of information organisations seek to know, it would be feasible to provide a rationale for which, if any, behavioural assessments might be used for this purpose. Additionally, such an understanding would provide the groundwork for future research to investigate whether knowledge of particular characteristics about a dog pre-adoption are associated with successful placement, which has received very limited research attention to date. A small amount of research has assessed whether particular behavioural characteristics present pre-adoption that have persisted post-adoption have an impact on placement success, but this has been tangential to studies' primary foci. In a study that investigated the relationship between food-related aggression observed pre-adoption using a standardised assessment and the presence of it post-adoption, it was found that even in dogs with owners that reported food-related aggression post-adoption, they did not consider it to be a significant problem (3). Future research with this as its primary focus would potentially improve the efficiency of organisations' pre-adoption screening processes, as they would not need to expend resources on gathering information that is not useful. Therefore, the aim of this study was to conduct a qualitative analysis of rehoming organisations' pre-adoption dog behaviour screening practices and review its evidence base. As part of this assessment, in addition to learning what types of information organisations aim to gather as part of their dog behaviour assessments, we investigated the weight / value given to this information and how it impacts on decisions relating to the rehoming of the dog. We then reviewed the evidence base for the factors identified.

## Materials and Methods

The information was gathered as part of a wider survey of rehoming organisations partly described in Griffin et al. (12). In brief, in the UK, a list of members of the Association of Dogs and Cats Homes (ADCH) was compiled in July 2012, which was used for participant recruitment in the current study; a total of 249 organisations and respective branches were contacted (comprised of 89 separate organisations, six of which had between two and 96 branches). Because data was collected for the current study following the completion of the related study Griffin et al. (12), additional resources were available to expand participant recruitment to rehoming organisations in the US. As such, a list of dog rehoming organisations in the US was compiled *via* the Petfinder website (www.petfinder.com/animal-shelters-and-rescues/) in August 2014. A total of 247 organisations were contacted (all separate organisations with no branches). The list of organisations that was compiled via the Petfinder website was also being used for participant recruitment in a related study involving in-person participation, so the eligibility criteria for organisations was geographical proximity to the principal investigator at the time (i.e., within a 60 mile radius). All organisations were only contacted electronically in a similar manner between February 2016 and July 2017. A combined total of 496 separate organisations and respective branches were contacted in the US and the UK. Organisations were asked about their pre-adoption dog assessments, such as those concerned with gauging temperament, personality, or behavioural characteristics. The current study sought to collect data about what information organisations aimed to gather about the dogs from the assessments, not what type of assessments they performed (if they used a formal or well-established one) [e.g., Match-Up Behaviour Evaluation (6), Canine-ality™ Assessment (www.aspcapro.org/resource/saving-lives-adoption-programs-behaviour-enrichment/what-canine-ality)]. Specific examples of pre-adoption behaviour assessments were not included in the written enquiry, as this may have discouraged organisations from responding who do not use a formal or well-established assessment in their screening process. Other pre-adoption dog assessments, such as veterinary checks, were not of interest. Specifically, organisations were asked:

“Do you assess the dogs in any manner prior to adoption, such as in terms of their temperament, personality, or behavioural characteristics?
If yes, are all dogs that are part of the organisation assessed?
  a. If no, why not?
Is there a form or document that is completed as a part of the assessment?
  a. If yes, would you be willing to please send…a copy of it?
What information about the dog (e.g., specific behaviours, personality characteristics, etc.) are you aiming to gather from the assessment? Please provide as much detail as possible.
5. Are any aspects of the assessment given more weight or value than others?
  a. If yes, what are they?
Would any results obtained from the assessment result in a dog being deemed unadoptable?
  a. If yes, what are they? Please provide as much detail as possible.
Is there anything else about the assessments of dogs conducted in your organisation that you would like to add?”

These questions were sent to organisations in the body of an email; responses could be submitted via email reply. They were given no specific guidelines or restrictions on how much to write in their responses, and the exact same written enquiry was sent to all organisations. They were also given the option to discuss their responses to the enquiry over the phone. Organisations were contacted twice electronically if they did not respond to the first contact attempt. All respondents were thanked for the information they provided and were not contacted any additional times for the purposes of this study.

A thematic analysis was undertaken using the procedural framework outlined by Braun and Clarke (13) to create the *data set*; this included only the information deemed relevant for analysis to achieve the current study's aims. Our aims are encapsulated in the answers found to the following four Research Questions:

**RQ1**. What information or characteristics about a dog are “most valued”? “Most valued” was defined as any characteristic reported to be given more weight in assessments, or to be sufficient for the dog to be deemed unadoptable.
**RQ2**. What information or characteristics about a dog would lead him/her to be deemed unadoptable? This included any evaluation with pass/fail criteria or characteristics stated to lead to a dog being deemed unadoptable (*sufficient criteria*).
**RQ3**. What evidence is in the scientific literature to support the inclusion of any of the characteristics as part of dog behaviour screening assessments? The scientific literature was reviewed to identify:
  1. Any statistically significant increased risks for relinquishment associated with the characteristic of interest. This focused on characteristics of surrendering owners and their dogs, and reasons reported by owners for surrendering their dogs. Those studies that reported purely descriptive relationships, i.e., not assessed statistically, were noted but were not included as scientific evidence.
  2. Whether the characteristic was significantly (in a statistical sense) associated with a dog's quality of life or overall welfare. This focused on published studies relating to owner and dog characteristics associated with a good quality of life for a dog in a non-clinical population (e.g., dogs that were not ill).
  3. Whether the characteristic could be associated with an increased risk to human safety. This focused on the scientific literature relating to the risk to humans of dog bites to any region of the body; specifically, studies evaluating owner-reported behavioural and management histories of dogs prior to biting incident(s). Those studies that reported purely descriptive relationships, i.e., not assessed statistically, were noted but were not included as scientific evidence.

A literature search was conducted online using various databases and search engines, such ScienceDirect, Wiley Online Library, and Google Scholar. Keywords used in this search included: *dog adoption, dog relinquishment, dog rehoming, shelter dogs, dog quality of life*, and *dog bites*. Articles that were not available online, such as for older publications, were accessed in journals' printed versions through the University of Lincoln library, or were requested via interlibrary transfer from The British Library. Studies published at any time were included in the search.

**RQ4**. What is the quality of the practical application of dog screening assessments?

This was addressed by assessing two aspects of the dataset: any reasons given for why not all dogs in an organisation were assessed, and why any information gathered but not reported to be highly valued, was being collected.

Data were organised using a “bottom up” or inductive approach (13). Information that appeared to be related and assessing the same constructs (e.g., a dog's behaviour around people) was grouped together to form a theme. The analysis further proceeded to create sub-themes, which were determined on the basis of three criteria:

1. the frequency of participants' responses regarding what information they aim to gather from assessments,
2. what respondents reported as the “most valued” information or characteristics (e.g., a dog's behaviour outside or in the garden), and
3. the presence of a characteristic in a dog that would lead him/her to be deemed unadoptable (e.g., a dog who bites). In this case, these characteristics may only have been stated by one organisation, but their necessity in the screening process and the significance of implications for a dog warranted them becoming a sub-theme in their own right.

Creating sub-themes was a multi-stage process that involved reviewing the data set multiple times at different points in the analysis. This was also necessary to deal with differences in semantics and terminology that could have led to redundancy in sub-themes. As such, sub-themes were added and subtracted as necessary. A two-tier structure of sub-themes emerged; with the tiers differentiated on the specificity of the characteristics. Themes encompassed general constructs, with the tiers of sub-themes progressively addressing more specific characteristics.

Ethical approval for this study was granted by the University of Lincoln College of Science Research Ethics Committee for research with human participants (approval number: UID CoSREC104).

## Results

Informative responses to the written enquiry were received from a total of 73 (14.69%) respondents (UK: *n* = 45, US: *n* = 28). Seventy-one responses (97.26%) were received via email reply. One response (1.37%) was given over the phone to the study's principal investigator, and one response (1.37%) was sent via post, even though this was not a response means provided in the electronic enquiry. All responses received by any means were included in the analysis. [Data was not collected about the specific nature or type of organisations that responded (e.g., size)]. Because the current study was exploratory in nature and was intended to provide an overview of what information rehoming organisations seek to gather from any type of pre-adoption dog behaviour assessment, the data collected from organisations in the UK and the US were analysed together.

Seventy-one respondents (97.26%) reported that they conduct some type of pre-adoption assessment on their dogs. Thirty-one respondents (43.67%) used some type of form in their assessments. Twenty-eight (39.44%) of the respondents provided relevant supplemental information, which was divided into two categories: dog assessment forms and surrendering owner forms. Dog assessment forms were those used by an organisation to conduct an assessment in terms of some aspect of the characteristics of the dog such as behaviour, temperament, or personality. The forms often included instructions for conducting the assessments and space to indicate how the dog performed in the assessment. Surrendering owner forms were those completed for dogs who were being relinquished to the organisation for rehoming. On these forms, the surrendering owner was asked a series of questions similar to those on the dog assessment forms. Non-relevant supplemental information provided by respondents included veterinary history and was excluded from the analysis. Eighteen respondents provided dog assessment forms; six provided surrendering owner forms; and four provided both forms. These were used in conjunction with respondents' answers to the questions on the written enquiry to address the four research questions.

### RQ1: What Information or Characteristics About a Dog Are “Most Valued”?

**Figure 1:**
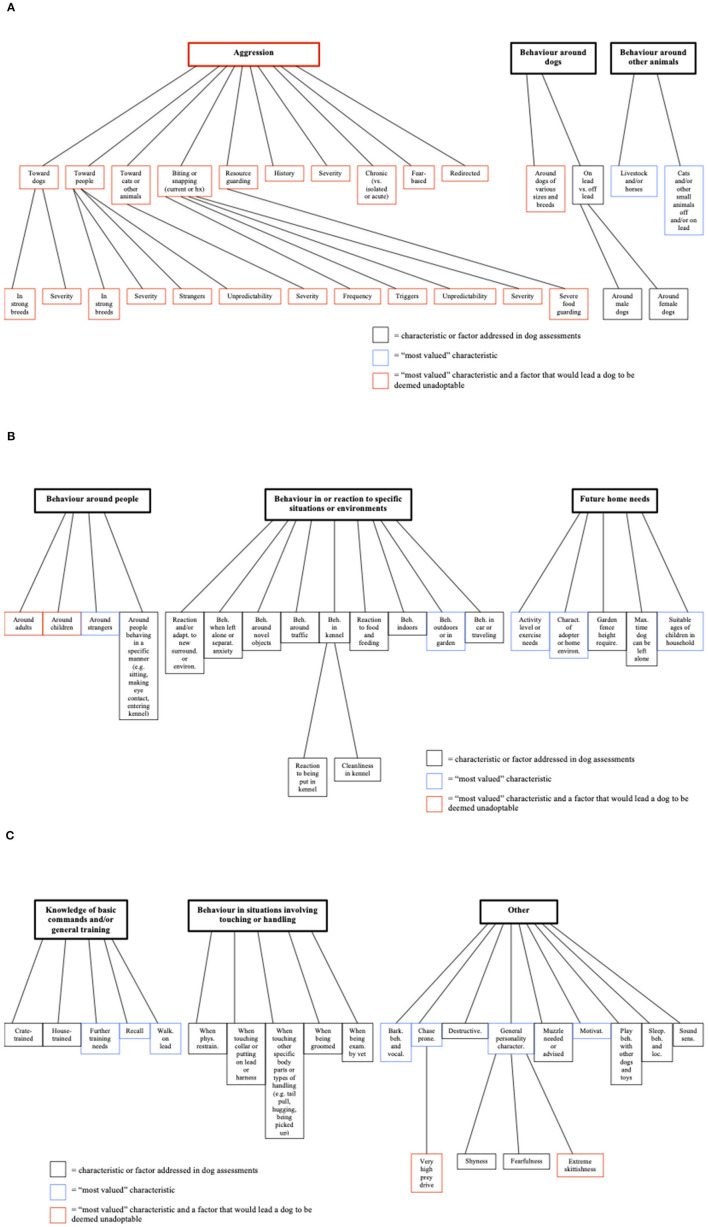
**(A–C)** Pre-adoption dog assessment themes and sub-themes.

Nine themes emerged from the analysis (see Table 1). Each theme and its sub-themes are illustrated in Figure 1. Due to variations in terminology and semantics, context was often used to parse and interpret underlying constructs and link sub-themes into themes. Identifying these underlying constructs was important for creating boundaries between the themes, i.e., criteria to differentiate one theme from another. This was particularly important for two themes, “*behaviour in or reaction to specific situations or environments*” and “*behaviour in situations involving touching or handling*”. The primary criterion used to differentiate the two was whether or not the dog is being physically touched or handled, often in a repetitive manner or over a period of time (e.g., while being groomed). Similarly, the theme “*aggression*” had overlapping characteristics with other themes (e.g., a dog's behaviour around people could be labelled as aggressive); it was made its own theme due to the overall emphasis on its reported importance. A total of 71 sub-themes were created within the nine themes (see Table 2).

**Table 1:** Themes present in pre-adoption dog behaviour assessments.

**Theme**	**Definition**
Aggression	Any type of dog behaviour that could be classified as potentially harmful or dangerous
Behaviour around dogs	A dog's behaviour in the presence of or towards another dog or dogs, which included purported evidence of sociability towards dogs, or lack thereof
Behaviour around other animals	A dog's behaviour in the presence of, or towards, another species
Behaviour around people	A dog's behaviour in the presence of, or towards, a person or people, which includes purported evidence of sociability towards people, or lack thereof
Behaviour in or reaction to specific situations or environments	A dog's behaviour when in specific situations that they may commonly experience in everyday life once rehomed (e.g., behaviour when traveling in a car)
Behaviour in situations involving touching or handling	A dog's behaviour in common situations that would involve him/her being touched or handled in a variety of ways by familiar and/or unfamiliar people (e.g., behaviour when physically restrained)
Future home needs	Aspects of a dog's future home, both in terms of adopter/family structure and the physical residence, that are deemed to be necessary for the dog (e.g., garden fence height)
Knowledge of basic commands and/or general training	Evidence, including report, of a dog performing basic commands (e.g., sit, stay, come) and/or other behaviours indicative of prior training (e.g., walking on lead behaviour)
Other	Miscellaneous sub-themes that were not relevant to the other themes, but were also not sufficient to create additional themes (e.g., sleeping behaviour and location)

**Table 2:** Number of sub-themes, tiers, and “most valued” dog characteristics by theme.

**Theme**	**Number of sub-themes**	**Number of tiers**	**Number of “most valued” characteristics**
Aggression^a^	22	2	23
Other	13	2	6
Behaviour in or reaction to specific situations or environments	11	2	1
Behaviour in situations involving touching or handling	5	1	0
Future home needs	5	1	3
Knowledge of basic commands and/or general training	5	1	3
Behaviour around dogs	4	2	1
Behaviour around people	4	1	3
Behaviour around other animals	2	1	2
TOTALS:	71		42

a *Aggression as a theme was a “most valued” characteristic itself*.

Forty-six respondents (64.79%) reported that they more highly value or give greater weight to some aspects of dog assessments than others. Eleven respondents (15.49%) reported that they did not, and this insight was not provided by 14 respondents (19.72%). Of the 71 sub-themes, 41 were rated as “most valued” (Figure 1). In addition, the theme “*aggression*” was a “most valued” characteristic in itself, with all 22 sub-themes contained in “*aggression*” also considered “most valued” characteristics, which was more than any other theme (Table 2). The theme “*other*” had the second most highly valued sub-themes; by contrast “*behaviour in situations involving touching or handling*” did not include any sub-themes that would lead a dog to being deemed unadoptable or reported as being highly valued.

Those themes that contain fewer of the “most valued” characteristics are not necessarily less important overall, but rather have fewer specific characteristics within the theme. For example, a respondent that rehomes greyhounds reported that, “The most significant characteristic in relation to Greyhounds [sic] is the ability to live with cats and other small animals. So, the ability to tolerate cats is the principal characteristic recorded.” This characteristic is clearly important to the respondent to assess, but it is broad in scope and is part of the “*behaviour around other animals*” theme, which had the fewest number of sub-themes.

### RQ2: What Information or Characteristics About a Dog Would Lead Him/Her to Be Deemed Unadoptable?

Being unadoptable does not necessarily mean that the dog will be euthanized; it refers to any other outcome aside from the dog being rehomed, such as being placed in a long-term foster situation or remaining part of the organisation indefinitely. Not all respondents reported that there are characteristics that would make a dog unadoptable, but for those that did, 49 respondents (69.01%) were identified as using a *pass/fail* assessment system, at least in part; 14 respondents (19.72%) did not use this sort of binary outcome, and it was unknown for eight respondents (11.27%). For respondents that used a *pass/fail* assessment system, some responses indicated that at least part of the reason for doing so was due to issues of public safety.

A total of 28 (66.7%) “most valued” characteristics were found to lead a respondent to deem a dog unadoptable (Figure 1). “*Aggression*” and all of its 22 sub-themes were the majority of the characteristics. The remaining five characteristics were part of three other themes: “*behaviour around dogs*”, “*behaviour around people*”, and “*other*”. Five of the themes did not contain any of these characteristics (Table 3).

**Table 3:** Number of characteristics that would lead a dog to be deemed unadoptable by theme.

**Theme**	**Number of characteristics**
Aggression	23
Behaviour around people	2
Other	2
Behaviour around dogs	1
Behaviour around other animals	0
Behaviour in or reaction to specific situations or environments	0
Behaviour in situations involving touching or handling	0
Future home needs	0
Knowledge of basic commands and/or general training	0
TOTAL:	28

### RQ3: What Evidence Is in the Scientific Literature to Support the Inclusion of These Characteristics as Part of Dog Behaviour Screening Assessments?

Evidence was found in the scientific literature to support 10 of the characteristics assessed (themes and sub-themes) from research that has examined reasons for relinquishment or characteristics of dogs who were relinquished and research that has investigated human safety risk: “destructiveness” (14–16), “housetrained” (15, 16), “activity level or exercise needs” (15, 16), “aggression towards people” (14, 16), “aggression towards cats or other animals” (16), “aggression towards dogs” (16), “biting or snapping” (15), “fearfulness” (15, 17), “resource guarding” (17), and “knowledge of basic commands and/or training” (18). The first seven characteristics listed were associated only with an increased risk for relinquishment. Two (“resource guarding” and “knowledge of basic commands and/or training”) were associated with only a risk to human safety, and one (“fearfulness”) was associated with both risks. No evidence could be found in the scientific literature to support the inclusion of any characteristics in assessments on the basis of a dog's quality of life or overall welfare. Of the 10 characteristics that were statistically associated with an increased risk of relinquishment and/or a risk to human safety, only five were characteristics that would lead a dog to be deemed unadoptable: “aggression towards people”, “aggression towards cats or other animals”, “aggression towards dogs”, “biting or snapping”, and “resource guarding” (Tables 4, 5). It is worth noting that in the published research, characteristics of dogs relinquished by surrendering owners were not necessarily the same as the reason(s) for relinquishment, and similarly, characteristics in owner-reported dog behavioural histories were not necessarily predictive of a human bite incident, but rather may just be associated with it.

**Table 4:** Dog assessment themes and sub-themes that are reported in the literature as reasons for relinquishment or characteristics of dogs as reported by surrendering owners.

**Reason/characteristic**	**Total number of studies mentioned in**	**Risk factor^a^**	**Number of studies with reported evidence^b^**	**“Most valued” characteristic^c^**	**Characteristic that would lead a dog being deemed unadoptable^c^**
Destructiveness	8 (14–16, 19–23)	√	3 (14–16)		
Housetrained	6 (15, 16, 19, 20, 22, 23)	√	2 (15, 16)		
Activity level or exercise needs	4 (15, 16, 20, 22)	√	2 (15, 16)	√	
Aggression towards people	4 (14, 16, 19, 22)	√	2 (14, 16)	√	√
Aggression towards cats or other animals	4 (16, 19, 20, 22)	√	1 (16)	√	√
Aggression towards dogs	4 (16, 19, 20, 22)	√	1 (16)	√	√
Biting or snapping	4 (15, 20, 22, 23)	√	1 (15)	√	√
Fearfulness	2 (15, 22)	√	1 (15)		
Barking behaviour and vocalization	5 (19–23)			√	
Aggression	3 (20, 21, 24)			√	√
Behaviour indoors	2 (20, 22)				
Behaviour outdoors or in garden	2 (20, 22)			√	
Very high prey drive	2 (20, 22)			√	√
Behaviour around cats and/or other small animals off and/or on lead	1 (21)			√	
Behaviour around dogs^d^	1 (21)				
Behaviour around children	1 (25)			√	√
Behaviour when left alone or separation anxiety	1 (20)				
Walking on lead behavior	1 (20)			√	

a *Reasons for which statistically significant evidence that the characteristics are associated with an increased risk of relinquishment*.

b *For an increased risk of relinquishment associated with each reason/characteristic*.

c *As reported by respondents in the current study*.

d *Mention in the literature was of the theme itself, not any of the “most valued” characteristics within the theme*.

**Table 5:** Dog assessment themes or sub-themes that are reported in the literature in owner-reported dog behavioural histories prior to biting incident.

**Reason/characteristic**	**Total number of studies mentioned in**	**Risk factor^a^**	**Number of studies with reported evidence^b^**	**“Most valued” characteristic^c^**	**Characteristic that would lead a dog to be deemed unadoptable^c^**
Fearfulness	2 (17, 18)	√	1 (17)		
Resource guarding	1 (17)	√	1 (17)	√	√
Knowledge of basic commands and/or general training^d^	1 (18)	√	1 (18)		
Aggression	1 (18)			√	√
Aggression towards dogs	1 (18)			√	√
Biting or snapping	1 (18)			√	√
Severity (of aggression)	1 (18)			√	√
Unpredictability (of aggression towards people)	1 (18)			√	√

a *Reasons for which statistically significant evidence that the characteristics are associated with an increased risk of relinquishment*.

b *For an increased risk of relinquishment associated with each reason/characteristic*.

c *As reported by respondents in the current study*.

d *Mention in the literature was of the theme itself, not any of the “most valued” characteristics within the theme*.

Five of the 10 characteristics for which evidence could be found in the literature to support their inclusion in assessments are part of the “*aggression*” theme; four of those were associated with an increased risk for relinquishment, and one (“resource guarding”) was associated with a risk to human safety. Even though minimal evidence was found in the literature to justify the inclusion of many of the characteristics that are part of the “*aggression*” theme, a concern of liability and a potential safety risk that could be associated with what might be construed as aggression or aggressive behaviours was reported by respondents in the current study. One organisation reported that they will not accept dogs for this reason, “*…we will not accept a dog that is showing aggressive behaviour as it is a safety risk for staff, volunteers and potential adopters*.” Three other organisations reported that it is why aggression is a reason for deeming dogs unadoptable,

- “*We do not believe that agressive [sic] dogs are safe for society. If training does not stop agessive behaviour [sic] we deem them unadoptable.”*
- “*We are aiming to ensure that the dog is, overall, safe to rehome*.”
- “…*if dog shows aggression that would make it unsafe to rehome responsibly.”*

Safety concerns over aggression towards people were reported, for example: “*Some behaviours determine that a dog cannot be safely rehomed…it is more difficult to safely rehome a dog displaying aggressive behaviour towards children*.” There are also safety concerns about aggression towards dogs or aggression towards cats or other animals. One organisation reported their concern about putting other animals in the home at risk, thus: “…*we would never put another dog or cat in danger if the dog in our care had aggressive tendencies towards other animals. It is not a successful placement unless all creatures in the house are comfortable and safe*.” Another organisation reported that they are particularly concerned with breed-related aggression towards dogs, as follows: “*We would have 'stricter' criteria with Strong Breeds [sic], including stricter criteria for dog-dog aggression*.” A third organisation reported that aggression towards dogs was a safety concern, and thus a characteristic that would lead to a dog being deemed unadoptable, by saying: “*If the dog is not good with other dogs and this is not manageable, modifiable or safe*.” Another organisation reported concerns about the potential risk associated with a dog who bites, as follows:

“*…if we think a dog can still be homed safely, we will do so. e.g., A Yorkshire Terrier who may bite is a lot different to a large/strong breed who may bite. So, too is the situation of when a bite may occur. e.g., A dog who bites with food guarding may still be adoptable Vs [sic] a dog who will bite when petted. Due to the nature of rescue, every effort will be made to find suitable homes for dogs. But' if they're deemed to be a danger to the public we will not re-home.”*

### RQ4: What Is the Quality of the Practical Application of Dog Screening Assessments?

Fifty-eight (81.69%) of the 71 respondents conducted assessments for all dogs pre-adoption. Three respondents (4.23%) did not assess all dogs, and this information was unknown for nine respondents (12.68%). The reasons provided by the three respondents for not assessing all dogs were:

- “*As far as formal assessments go, they are not necessary for the majority of the dogs we take in because we are foster based and get to know the dogs so well in our homes, but our trainer will give us a full assessment (which we pay for) so we know how best to work with any issues we may observe. So far we have used our trainer to formally assess and work with two of our dogs, both pitbulls*.”
- “*Manpower, finance and weighing up the actual need to temp test every dog*.”
- “*We rely on trusted shelter staff occasionally*.”

While the first two responses provide a clear rationale, the third response is less clear. The respondent may have been implying that they have a limited number of shelter staff who are sufficiently trained to conduct assessments, which would then mean that a lack of resources is the issue.

As previously noted, 64.79% (46/71) of respondents reported that they highly value or give more weight to certain characteristics in dog assessments. For the 15.49% (11/71) of respondents who reported that they do not highly value certain characteristics or criteria, they provided responses about their alternative assessments. Sample responses included:

- “*No everything is taken into consideration*.”
- “*No—more would depend on the potential home and to how suitable they were for the particular dog*.”
- “*No—it is all just as important to ensure the dog is happy, given the correct support and finds the right home. All info is needed to get a full picture*.”

These responses suggest that one reason why respondents address so many characteristics in assessments, even if they are not highly valued, is because they are aiming to acquire a much more general or complete view of the dog in the hope that this will allow them to more accurately match the dog to an appropriate adopter. Likewise, one respondent who reported that aggression towards humans is more highly valued, also stated with regard to the rest of the characteristics included in assessments: “*Other areas are designed more for information purposes/matching dogs up with suitable owners*.” Another respondent that reported a dog's behaviour around humans is more highly valued, also stated that: “*The assessment acts as an overall guide to build a picture, often elements link*.” Collectively all of the responses suggest, regardless of whether a respondent does or does not more highly value certain characteristics or criteria, there is an emphasis on a “whole picture approach” to assessing dogs, which focusses on gaining as much information as possible about a dog from assessments.

## Discussion

Although many respondents (71/73) assess all dogs pre-adoption, the key reasons for not doing this seem to be a lack of resources and the belief that not all dogs need to be assessed (e.g., due to their breed). Many respondents (46/71) included assessment of less valued characteristics, and this appeared to be for wider informational purposes to improve future matching of a dog to an adopter. However, in the absence of any quality assessment of the reliability of these processes, it needs to be recognised that they may be putting an unnecessary strain on their resources.

The scientific literature only supported “destructiveness”, “housetrained”, “activity level or exercise needs”, “aggression towards people”, “aggression towards cats or other animals”, “aggression towards dogs”, “biting or snapping (current or history)”, “fearfulness”, “resource guarding”, and “knowledge of commands and/or general training” as risk factors for relinquishment and/or risk factors to human safety, with five of them (“aggression towards people”, “aggression towards cats or other animals”, “aggression towards dogs”, “biting or snapping”, “resource guarding”) leading to a dog being deemed unadoptable by some organisations (Tables 4, 5). Although the risk of relinquishment increases with the frequency of “destructiveness”, “housetrained”, and “fearfulness”, and the risk associated with these behaviours are mentioned in multiple studies (14–16, 19–22), it is perhaps surprising these were not widely considered to be “most valued” factors. For example, in the case of housetraining as a risk factor for relinquishment, Patronek et al. (16) reported that dogs who were reported to have had inappropriate elimination ≤2 times per month had a 1.46 times (95% CI: 1.01-2.11) increased risk for relinquishment. The risk increased to 3.36 (95% CI: 2.09-5.38) for those who had inappropriate elimination weekly, and it increased further to 8.52 (95% CI: 5.23-13.87) for those who had inappropriate elimination on a daily basis. Similarly, New et al. (15) reported that dogs who were reported by their surrendering owner to have soiled inside the house “some of the time” had a 1.2 times (95% CI: 1.1-1.4) increased risk for relinquishment. Those who soiled inside the house “most of the time” had a 2.7 times (95% CI: 2.1-3.7) increased risk for relinquishment, and those who soiled inside the house “always/almost always” had a 3.7 times (95% CI: 2.7-4.9) increased risk. It is possible that housetraining issues were more frequently reported for dogs at ages when housetraining issues might be more likely to occur (e.g., puppies). It also might be that dogs who were left home for extended periods of time had more issues with housetraining. It would be useful for future research to investigate possible relationships between such factors and housetraining issues. Having said that, it is possible that organisations are more concerned with human safety risk and overall liability than with relinquishment risk, so they may give greater importance to characteristics that could pertain to public safety (e.g., those under the “*aggression*” theme). Therefore, organisations may not be as concerned with factors, such as housetraining issues, that have been found to be associated with an increased risk for relinquishment that do not also pose a public safety risk. It is also notable that “activity level or exercise needs” is mentioned in multiple studies (15, 16, 20, 22), and two of them (15, 16) provide evidence for an increased risk for relinquishment associated with this issue. New et al. (15) reported that the relinquishment risk associated with this characteristic increases with the frequency of the displayed behaviours. Furthermore, just as many studies reported that this characteristic was associated with an increased risk for relinquishment as for the characteristics under the “*aggression*” theme. A possible explanation for this is that surrendering owners may be over-reporting hyperactivity, as they might believe it to be more socially acceptable than anything related to aggression. Another plausible explanation is that in general organisations may be less likely to admit dogs who have histories of or display any behaviours that could be construed as aggression or that such dogs are privately euthanized due to safety risk, whereas hyperactive dogs could be seen as having more potential for rehabilitation and thus could be easier to rehome. This deserves further investigation.

The theme of “*aggression*” contains many (23/42) of the “most valued” characteristics, all of which would lead a dog to be deemed unadoptable. It is clear that screening for aggression, or what might be characterised as aggressive behaviours, is a central focus in organisations' dog assessments; however, the predictive value of such tests is unknown, and even for published tests it is often poor (26). Scientific evidence could be found to support the importance of five of these in increasing the risk of relinquishment (“aggression towards people”, “aggression towards dogs”, “aggression towards cats or another animals”, and “biting or snapping”). Specific scientific evidence could be found to support an additional one of these characteristics (“resource guarding”) on the basis of an increased risk to human safety. We summarise this evidence below. Patronek et al. (16) reported that dogs who were aggressive towards people on a weekly basis were 2.41 times (95% CI: 1.44-1.03) more likely to be relinquished; by contrast, those who were aggressive on daily basis had a 2.14 (95% CI: 1.25-3.66) increased risk. Diesel et al. (14) reported that dogs who displayed aggression towards people were at a statistically significant greater risk for return compared to dogs who did not display aggression towards people, but the risk level varied based on owners' response to it. Compared to dogs without behavioural problems, those who displayed aggression towards people and had owners who sought advice were 5.6 times (95% CI: 3.4-9.4) more likely to be returned, while those who had owners who did not seek advice had 11.1 times the risk (95% CI: 6.6-18.8). New et al. (15) reported that dogs who had bitten a person were 2.9 times more likely to be relinquished (95% CI: 2.4-3.6). Guy et al. (17) reported that biting dogs were 3.08 times more likely to have shown aggression over food^1^ in the first 2 months of ownership (95% CI: 1.05-9.01). In relation to aggression towards other species, Patronek et al. (16) reported that dogs who were aggressive towards other pets on a daily basis had a 2.91 times (95% CI: 1.57-5.39) increased risk for relinquishment. We considered “aggression towards dogs” and “aggression towards cats or other animals” as separate “most valued” characteristics. However, the literature reviewed seemed to fail to make this distinction, referring to aggression towards pets or aggression towards animals, with the two discussed together here. Respondents in the current study did not specify whether they were referring to aggression towards dogs and animals within the household or aggression towards them in general. Aggression towards animals within the household is a different risk to aggression towards animals outside of the household, but that potential differentiation is outside the scope of the current study.

For the majority of the characteristics under the “*aggression*” theme, no specific research evidence could be found in the scientific literature to support their inclusion in assessments on the basis of increased relinquishment risk or human safety risk, although several more were at least mentioned in the literature [e.g., “very high prey drive” (20, 22)] (Table 6). However, it is possible that at least some of these characteristics are risks factors, but they have just not been investigated in the literature to determine this (i.e., absence of evidence is not evidence of absence). One reason for this may be due to the specific nature of some of the characteristics. In the case of “very high prey drive,” it is a characteristic that is often associated with specific breeds (e.g., greyhounds). However, without engaging breed specific rehoming organisations in relevant research, the scientific data will be limited in its scope and relevance to the full constituency of those involved in shelter and rehoming work. The lack of evidence for many characteristics may also be due to discrepancies in semantics between how respondents described what types of information they aim to gather from assessments in the current study and what are reported as risk factors in the scientific literature. This potential issue could be addressed and clarified in future research by gathering data in a forced-choice manner based on the data generated here rather than the open-ended manner required in a pioneering study such as this.

**Table 6:** Dog assessment sub-themes (characteristics) for which there is no evidence^a^ in the literature to support their inclusion in pre-adoption behaviour assessments.

**Sub-themes**	**“Most valued” characteristic^b^**	**Characteristic that would lead a dog to be deemed unadoptable^b^**
Aggression towards dogs in strong breeds	√	√
Severity of aggression towards dogs in strong breeds	√	√
Aggression towards people in strong breeds	√	√
Severity of aggression towards people	√	√
Severity of aggression towards cats or other animals	√	√
Aggression towards strangers [people]	√	√
Unpredictability of aggression towards people	√	√
Frequency of biting or snapping (current or history)	√	√
Triggers for biting or snapping (current or history)	√	√
Unpredictability of biting or snapping (current or history)	√	√
Severity of biting or snapping (current or history)	√	√
Chronic aggression (vs. isolated or acute)	√	√
Fear-based aggression	√	√
History of aggression	√	√
Redirected aggression	√	√
Severe food guarding	√	√
Severity of aggression	√	√
Behaviour around adults	√	√
Behaviour around children	√	√
Behaviour around dogs of various sizes and breeds	√	√
Extreme skittishness	√	√
Very high prey drive	√	√
Barking behaviour and vocalization	√	
Behaviour around cats and/or other small animals off and/or on lead	√	
Behaviour around livestock and/or horses	√	
Behaviour around strangers [people]	√	
Behaviour outdoors or in garden	√	
Characteristics of adopter or home environment	√	
General personality characteristics [of dog]	√	
Chase proneness	√	
Motivation [of dog]	√	
Further training needs	√	
Recall [proficiency of]	√	
Suitable ages of children in household	√	
Walking on lead [proficiency of behaviour]		
Behaviour around dogs on lead vs. off lead		
Behaviour around male dogs on lead vs. off lead		
Behaviour around female dogs on lead vs. off lead		
Behaviour around novel objects		
Behaviour around traffic		
Behaviour in car or traveling		
Behaviour indoors		
Behaviour when being examined by a vet		
Behaviour when being groomed		
Behaviour when physically restrained		
Behaviour when touching collar or putting on lead or harness		
Behaviour when touching other specific body parts or types of handling (e.g., tail pull, hugging, being picked up)		
Maximum time dog can be left alone		
Behaviour in kennel		
Reaction to being put in kennel		
Cleanliness in kennel		
Behaviour around people behaving in a specific manner (e.g., sitting, making eye contact, entering kennel)		
Behaviour when left alone or separation anxiety		
Crate-trained		
Garden fence height requirement		
Muzzle needed or advised		
Play behaviour with other dogs and toys		
Reaction and/or adaptability to new surroundings/environment		
Reaction to food and feeding		
Shyness		
Sleep behaviour and location		
Sound sensitivity		

a *No statistically significant association between the characteristic and an increased risk for relinquishment, a dog's quality of life or overall welfare, or an increased risk to human safety was reported in the literature*.

b *As reported by respondents in the current study*.

This study was intended to provide an overview of what information rehoming organisations seek to gather from pre-adoption dog behaviour assessments, regardless of by what means they gather it. Although a considerable body of literature has investigated the usefulness of specific behaviour assessments [e.g., (3, 9)], including the development of new assessments [e.g., (27)], little is known about what types of information about a dog organisations aim to gather from any type of assessment. Therefore, due to its exploratory purpose, the current study pooled the data collected from all organisations and analysed it together. We recognise that in general organisations in the UK may be fundamentally different from those in the US, so investigating whether there are any differences between practices in the two countries would be a useful direction for future research. The fact that the data was pooled in the current study may have not allowed for differences in responses between the two countries to be recognised. Similarly, the current study did not collect information about the nature of participating organisations (e.g., size, municipal vs. private, urban vs. rural setting), which may have been a limitation of the study. Therefore, gathering this type of information in future research would be useful to determine if differences in the nature of an organisation are associated with differences in dog behaviour screening practices. An additional limitation of this study may have been the location of participating organisations within each country. Those in the UK were located throughout the country, whereas those in the US were located in relatively close proximity to each other in a specific region of the country. A more targeted participant recruitment approach in future research would help to mitigate the location biases in the current study.

The results of the current study indicate vast amounts of information are gathered by shelters and rehoming organisations as part of pre-adoption dog assessment. Aside from the lack of evidence in the scientific literature to justify the inclusion of the majority of the characteristics they seek to gather information about (Table 6), there is also the issue, as previously noted, of the quality of the assessments themselves. Several studies have evaluated the reliability and validity of dog assessments (30), and often with a focus on screening for aggression. In an evaluation of a standardised temperament test used to assess dogs pre-adoption, Christensen et al. (2) reported that there are certain types of aggressive behaviour (i.e., territorial, predatory, and intra-specific) that are not reliably exhibited during the test, and thus the test poorly identifies these types of aggression. Marder et al. (3) found that a different standardised dog behaviour assessment was better at predicting an absence of food-related aggression post-adoption than the presence of it. Consequently, the authors concluded that the detection of food-related aggression in a behaviour evaluation should be interpreted with caution, and the presence of such behaviours pre-adoption are not necessarily predictive of post-adoption behaviour. Poulsen et al. (9) evaluated the predictive validity of another behavioural assessment used to screen dogs pre-adoption, and found that it was unable to predict specific behaviours, such as aggressive tendencies towards conspecifics and escaping tendencies, which suggests that the assessment is not particularly useful for its intended purpose. In an investigation of behaviour assessments used by several shelters, Mornement et al. (1) found that many assessments developed in-house are widely used and they lack standardisation in content and methodology, and none had been adequately evaluated in the scientific literature. The study also reported that while some shelters may use assessments for which there has been scientific validation established, they modify them to fit their needs and/or have not received adequate training for administration, so they may no longer be valid. Based on the reports of such studies, the usefulness of the vast majority of dog assessments is debatable, so perhaps there should be a shift in organisations' focus and resources away from dog assessments, the outcomes of which can often have grave consequences for dogs. This argument has been put forth by Patronek and Bradley (10) and Patronek et al. (11), who have suggested a shift away from the usage of dog assessments in shelters due to assessments' lack of predictive value (i.e., assessments are unable to predict problematic behaviour in a home). Alternatively, it might be much more beneficial for organisations to instead focus resources on educating owners pre-adoption and supporting adopters post-adoption, for which there is evidence in the literature that this significantly increases the likelihood that a dog will remain in a home and not be relinquished (14, 16). Research has found that inappropriate expectations or a mismatch in expectations, such as with the amount of work required to care for a dog, can increase the likelihood that a dog will be relinquished (15, 16), so educating adopters prior to adoption about various aspects of dog ownership may well increase the likelihood that the placement will be successful. It is recognised that even if rehoming organisations do offer post-adoption support, that does not necessarily mean that adopters will take advantage of the resources available to them, even if they are aware of their existence (28). Research is therefore needed to evaluate how to better support adopters post-adoption and to encourage them to use the resources that are available. It is possible that there are numerous reasons why adopters do not take advantage of such resources, and the reasons could vary widely due to a number of factors, such as location. Until more is known about how to encourage adopters to use available supports and resources, it might be worthwhile for organisations to focus on keeping in touch with adopters post-adoption, at least for the first year (15), so that if the relationship begins to break down, such as if a behavioural issue arises or an unforeseen life event happens, a line of communication has already been established. It is possible that by the time an issue arises that could cause an owner to consider relinquishment, they are already in a state of crisis and are unreceptive to support, at which point it may be more difficult to intervene.

## Conclusion

Screening for potential behavioural issues is clearly central to assessments, and they are frequently reported reasons for relinquishment and/or behaviours displayed by dogs who have bitten a human. However, there is statistically significant evidence of an increased risk for relinquishment or human safety risk associated with <15% of these features, and no evidence could be found in the literature of an association between any of these behaviours and a dog's quality of life. This is clearly a significant gap in the research literature. The evidence pertaining to relinquishment risk and human safety risk offers justification for including screening for some behaviours in assessments, and it highlights the gravity of the role behavioural issues can have in the breakdown of the dog-owner relationship. However, it is worth noting that there is very limited evidence concerning the predictive validity of any in-house behavioural tests [e.g., (1, 9)], and while such information may be provided by owners surrendering their dog, it is likely that they do not provide a full and complete behavioural record of their pet. It is striking that the number of characteristics that would lead a dog to be deemed unadoptable is nearly three times the number of characteristics with reported evidence of an increased risk of relinquishment. Moreover, only five of the characteristics that would cause a dog to be deemed unadoptable (“aggression towards people”, “aggression towards cats or other animals”, “aggression towards dogs”, “biting or snapping”, and “resource guarding”) are reported risk factors for relinquishment or risk factors to human safety. The remaining 23/28 of the characteristics could cause a dog to be labelled unnecessarily as unadoptable, and depending on the organisation, this could have profound consequences for the dog.

It is possible that some of the motivation behind gathering particular information in dog screening assessments is to ensure a good quality of life for the dog. However, assessing and predicting a dog's quality of life is very challenging as it should encompass both deliberate suffering and unconscious inadequate care. There is little literature relating to the assessment of quality of life in non-clinical populations [e.g., (29)], and this is clearly challenging to undertake.

An initial step that future research could take in this direction would be to build a consensus on what qualifies as a successful placement and what it looks like in practice. From there, the potential ways in which to objectively measure success, and ultimately quality of life, could be explored in order to ensure dogs are treated consistently. Equally important is the need for prospective longitudinal research to thoroughly evaluate the overall usefulness of pre-adoption behavioural evaluations to the success of a dog placement, with a specific focus on the characteristics reported as highly valued in the current study. As was previously noted, it is possible that many more of the characteristics included in pre-adoption behaviour screenings are associated with an increased risk to human safety, an increased risk for relinquishment, or affect a dog's quality of life, but these specific relationships have not yet been investigated. Because all characteristics under the “*aggression*” theme were reported as “most valued” in this study and could lead a dog to being deemed unadoptable, particular research attention should be paid to assessing potential associations between those and human safety risk, relinquishment risk, and quality of life. Perhaps instead of focusing on investigating the predictive validity of individual behaviour assessments, it would be more beneficial to compare post-adoption behaviour of dogs who have been behaviourally screened pre-adoption (using any assessment or by any means) with those who have not been. This could provide more insight into the overall value of dog behaviour screenings. Although some studies have had a prospective study design when investigating various aspects of dog behaviour assessments and failed dog placements [e.g., (3, 14)], the duration for which they follow dogs post-adoption needs to be increased in order to have more meaningful results. Dogs remain at an increased risk for relinquishment for the first year after acquisition (15), so prospective longitudinal research should continue to track placement outcomes for at least one year post-adoption. Until additional research is conducted to evaluate potential relationships between the specific types of information sought in pre-adoption assessments and relinquishment risk, human safety risk, or a dog's quality of life, and because the usefulness of the vast majority of dog assessments is debatable, organisations should shift their focus and resources to educating owners about dog ownership pre-adoption and post-adoption support, especially to keep the lines of communication open in case an issue that could lead to relinquishment arises.

## Data Availability Statement

The raw data supporting the conclusions of this article will be made available by the authors, without undue reservation.

## Ethics Statement

The studies involving human participants were reviewed and approved by University of Lincoln Ethics Committee Lincoln, United Kingdom. The patients/participants provided their written informed consent to participate in this study.

## Author Contributions

KG and DM determined the rationale for and the necessity of this study to the field and decided on the methods for the study. KG collected the data from the participants and conducted most of the data analysis, the literature review, and wrote the initial draft of the paper. DM contributed to the data analysis, reviewed multiple drafts of the paper, and provided substantiative feedback and editing, including highlighting sections of the paper that needed revising. TP and EJ assisted with the structure of the paper and edited multiple drafts of it, including providing feedback on the methods of data analysis used. All authors contributed to the article and approved the submitted version.

## Funding

Funding for the open access publication of this study has been provided by Maddie's Fund^®^.

## Conflict of Interest

The authors declare that the research was conducted in the absence of any commercial or financial relationships that could be construed as a potential conflict of interest.

## Publisher's Note

All claims expressed in this article are solely those of the authors and do not necessarily represent those of their affiliated organizations, or those of the publisher, the editors and the reviewers. Any product that may be evaluated in this article, or claim that may be made by its manufacturer, is not guaranteed or endorsed by the publisher.

## Footnotes

^1^In the current study, we classified *aggression over food* as a type of resource guarding.
